# Attention shifts the language network reflecting paradigm presentation

**DOI:** 10.3389/fnhum.2013.00809

**Published:** 2013-11-25

**Authors:** Kathrin Kollndorfer, Julia Furtner, Jacqueline Krajnik, Daniela Prayer, Veronika Schöpf

**Affiliations:** ^1^Department of Biomedical Imaging and Image-guided Therapy, Medical University of ViennaVienna, Austria; ^2^Department of Neurosurgery, Medical University of ViennaVienna, Austria

**Keywords:** fMRI, language, attention-shift network, functional mapping, visual, auditory

## Abstract

**Objectives:** Functional magnetic resonance imaging (fMRI) is a reliable and non-invasive method with which to localize language function in pre-surgical planning. In clinical practice, visual stimulus presentation is often difficult or impossible, due to the patient's restricted language or attention abilities. Therefore, our aim was to investigate modality-specific differences in visual and auditory stimulus presentation.

**Methods:** Ten healthy subjects participated in an fMRI study comprising two experiments with visual and auditory stimulus presentation. In both experiments, two language paradigms (one for language comprehension and one for language production) used in clinical practice were investigated. In addition to standard data analysis by the means of the general linear model (GLM), independent component analysis (ICA) was performed to achieve more detailed information on language processing networks.

**Results:** GLM analysis revealed modality-specific brain activation for both language paradigms for the contrast visual > auditory in the area of the intraparietal sulcus and the hippocampus, two areas related to attention and working memory. Using group ICA, a language network was detected for both paradigms independent of stimulus presentation modality. The investigation of language lateralization revealed no significant variations. Visually presented stimuli further activated an attention-shift network, which could not be identified for the auditory presented language.

**Conclusion:** The results of this study indicate that the visually presented language stimuli additionally activate an attention-shift network. These findings will provide important information for pre-surgical planning in order to preserve reading abilities after brain surgery, significantly improving surgical outcomes. Our findings suggest that the presentation modality for language paradigms should be adapted on behalf of individual indication.

## Introduction

Brain surgery that involves eloquent cortical areas, particularly in brain tumor or epilepsy patients, has remained a challenging task (Spena et al., 2010). Preservation of neuronal functions after surgery is one of the most important goals for neurosurgeons. An accurate mapping of eloquent cortical areas ensures a sufficiently extensive and safe resection of brain parenchyma. Functional magnetic resonance imaging (fMRI) has been established as a reliable and noninvasive tool in mapping of cognitive and executive functions prior to brain surgery [for review see Dimou et al. (2013)]. Reliable localization of language abilities is of huge importance in pre-surgical planning, as language is an essential quality of life factor. The gold standard for intraoperative language localization and neuronavigation is direct electrocortical stimulation (ECS; Sunaert, 2006). However, this method is time-consuming during surgery and is not applicable in all cases, as compliance of the awake patient during surgery is mandatory, and not all patients are capable of this.

Patients who undergo fMRI examination prior to neurosurgery often suffer from disease-driven restricted language abilities or have difficulties in focusing their attention on the task for the entire measurement period. Reading is especially challenging for patients undergoing pre-surgical planning, and therefore, stimuli are often presented auditorily to map language abilities (Dimou et al., 2013). However, the manner in which stimuli are presented might influence the spatial representation of processing networks, as already hypothesized by Carpentier et al. (2001), who investigated differences between auditory and visual stimulus presentation in language-related areas.

In clinical practice, two different language paradigms, one for language perception and one for language production are usually presented visually to map language-related areas. The present study aimed to investigate the different processing networks related to presentation modalities of these exact paradigms by testing auditory and visual stimulus conditions. Based on previous findings and clinical observations that visually presented fMRI stimuli are particularly challenging for patients, we were interested in the specific characteristics of networks that process written language. Therefore, we hypothesized that visually presented language stimuli would require an attention-shift network (Corbetta et al., 1998; Corbetta and Shulman, 2002) in the brain.

To achieve a conclusive comparison of both techniques, two different analysis approaches were used to account for temporal and spatial network patterns: data driven analysis was performed, using independent component analysis (ICA), to test for functionally connected processing networks; and a hypothesis-driven method, using a general linear model (GLM), was used to account for purely stimulus-driven activity. Combining these two analysis methods offers complementary information about the precise processing and representation of language-related areas. As the shift of attention induced by different stimulus modalities is not clear yet, ICA is an appropriate method to investigate data without assuming an *a priori* model, as this method discriminates activation based on spatial independence rather than temporal correlation to a predefined stimulus.

## Materials and methods

### Subjects

Ten healthy right-handed subjects (four male, six female; mean age 22 years) participated in this study. All participants completed two fMRI experiments, comprising two scanning sessions each: Experiments 1 and 2. All subjects had normal or corrected-to-normal vision and no history of psychiatric or neurologic diseases. All participants were native speakers of the German language and had a comparable educational background. Prior to inclusion, all participants were informed about the aim of the study and gave their written, informed consent. The study was approved by the Ethics Committee of the Medical University of Vienna.

### Behavioral data

To avoid influence of language abilities on neural activation within the language network, two language tasks were performed prior to fMRI measurements. The first task was a sentence completion task, a subtest of the Intelligence Structure Test (IST-2000-R; Liepmann et al., 2007), which tests for semantic decision-making. This subtest consists of 20 sentences that are missing the last word of the sentence. The participant is instructed to choose one of five given words to complete the sentence correctly. Furthermore, all participants completed the Regensburg Word Fluency Test (RWT; Aschenbrenner et al., 2000), which tests for verbal fluency, and reflects semantic memory. Subjects had to pronounce as many words as possible referring to a given category. This category can be semantic, such as fruits or animals, or phonemic, such as words beginning with the letter *M (e.g., mother, man, mouse)*.

### Experiment 1

In Experiment 1, subjects were visually presented with two different language paradigms using an MR-compatible visual stimulation system (NordicNeuroLab, Bergen, NO).

1. Verb generation task: The first language paradigm was a covert verb generation task. Frequent German nouns were visually presented in white letters on a black screen. In this task, a 30s block-design was used. During active blocks, 15 nouns are presented for 1s each (e.g., door, book, ball). The subjects were instructed to think of all verbs he/she associated with the presented noun until the next word appeared (Petersen et al., 1988; Holland et al., 2001). During baseline blocks, the participants were asked to fixate on harsh signs presented on the screen.
2. Phrases task: In the second language paradigm, syntactically simple and correct sentences in canonical German word order (subject–verb–object) were presented in white letters on a black screen. During active blocks, sentences were presented every 2 s, half of the sentences containing a semantically inappropriate object *(e.g., semantically appropriate: Das Mädchen spielt Klavier. Engl.: ’The girl plays the piano.'; semantically inappropriate: Der Dichter dichtet ein Auto. Engl:. ’The poet composes a car.')*. During baseline, subjects were instructed to look at white harsh signs presented on the black screen [stimuli modified from Foki et al. (2008)].

### Experiment 2

Experiment 2 consisted of the same two language paradigms. Rather than visual presentation, words and sentences were presented auditorily using MR-compatible head phones. Block-design presentation times equaled those of Experiment 1. During active blocks, 15 nouns or sentences were presented. During baseline, participants were presented with a tone every 2 s.

### Imaging methods

Measurements were performed on a 3T TIM Trio System (Siemens Medical Solution, Erlangen, Germany) using a 12-channel head coil. FMRI data were acquired using single-shot, gradient-recalled, echo-planar imaging (EPI). Twenty slices (1 mm gap, 4 mm thickness) with an FOV of 210 × 210 mm and a TE/TR of 42/2000 ms were acquired. Slices were aligned parallel to the connection between the anterior and posterior commissure. All subjects participating in this study underwent four scanning sessions, two with visually presented language paradigms (Experiment 1) and two with auditory language presentation (Experiment 2), lasting 5 min each.

Stimulus fixation and eye movements were recorded using an MR-compatible eye-tracker (ViewPoint EyeTracker, Arrington Research, Scottsdale, AZ) throughout all the measurements of Experiment 1.

### Data analysis

Preprocessing of fMRI data was performed using SPM8 (http://www.fil.ion.ucl.ac.uk/spm/) implemented in MATLAB (Matlab 7.14.0, Release 2012a, Mathworks Inc., Sherborn, MA, USA) including motion correction, spatial normalization to an MNI template, and spatial smoothing. First-level analysis was performed for each paradigm separately, by constructing a GLM using block onsets as regressors. Head movement effects were modeled by including six motion parameters as additional regressors. The contrast active > baseline was generated for both paradigms for Experiments 1 and 2. For comparison of visually vs. auditorily presented language effects, the two contrasts visual > auditory and auditory > visual were calculated at the group level.

Additional second-level group analysis was carried out for both paradigms (phrases and verb generation) and experiments (auditory and visual presentation) using probabilistic ICA, as implemented in MELODIC (Multivariate Exploratory Linear Decomposition into Independent Components) version 3.10, a part of FSL (FMRIB's Software Library, www.fmrib.ox.ac.uk/fsl), using FastICA (Beckmann and Smith, 2004). Non-brain voxels were masked and voxel-wise de-meaning of the data and normalization of the voxel-wise variance was carried out. Pre-processed data sets were whitened and projected into an n-dimensional subspace using probabilistic Principal Component analysis in which the number of dimensions was estimated using the Laplace approximation to the Bayesian evidence of the model order (Minka, 2000; Beckmann and Smith, 2004). Dimensions for n were 18 for both visually presented paradigms, 24 for the auditory phrases task, and 25 for the auditory verb generation task. For the optimization of the non-Gaussian sources, contrast function and convergence thresholds, as suggested by Hyvärinen et al. (2001), were used. Estimated component maps were divided by the standard deviation of the residual noise and thresholded by fitting a mixture model to the intensity values histogram (Beckmann and Smith, 2004). All group ICA network components were assessed by visual inspection, based on the spatial distribution patterns.

Additional group ICAs were carried out by submitting the visual and auditory data sets of both conditions to be evaluated as a group. After group ICA, as described above, the set of spatial maps from the group-average analysis was used to generate subject-specific versions of the spatial maps, and associated time series, using the dual regression approach version v0.5, a part of FSL (Beckmann et al., 2009; Filippini et al., 2009). First, for each subject, the group-average set of spatial maps is regressed (as spatial regressors in a multiple regression) into the subject's 4D space-time dataset. This results in a set of subject-specific time series, one per group-level spatial map. Next, those time series were regressed (as temporal regressors, again in a multiple regression) into the same 4D dataset, resulting in a set of subject-specific spatial maps, one per group-level spatial map. Corresponding spatial IC maps for every subject and both conditions were then exported to SPM8 for statistical testing. For second-level analysis, two separate *t*-tests were performed for both conditions (*p* < 0.05, FWE corrected). Common language related areas, independent of presentation modality, were investigated performing two conjunction analyses (Friston et al., 1999), one for visual and auditory presentation of the phrases task and a second one for the two modalities of the verb generation task (*p* < 0.05, FWE corrected).

To investigate language lateralization, voxel-wise laterality maps were created for the subject-specific spatial IC maps resulting from the dual regression step. The lateral maps were computed using the LUI toolbox (http://mialab.mrn.org/software/; Swanson et al., 2011) by subtracting every image from itself after flipping in the left/right direction (Stevens et al., 2005). A voxel-wise laterality map overcomes the problem of a laterality index, which is based on voxel counting and is therefore sensitive to the definition of the threshold. Two-sample *t*-tests were calculated across the two presentation modalities separately for both tasks (*p* < 0.05, FWE corrected).

## Results

### Behavioral data

The results of the language tasks performed prior to fMRI measurements revealed average language performance for all investigated subjects. For the sentence completion subtest, the participants' number of correct items ranged from 10 to 18 (mean 14), corresponding to an average performance compared to normative data for this age group. Results of the RWT revealed a mean number of 18 words beginning with the letter *M* and a mean number of listed words referring to the category of animals of 35, both reflecting average verbal fluency performance.

### Hypothesis-driven analysis (GLM)

**Figure 1:**
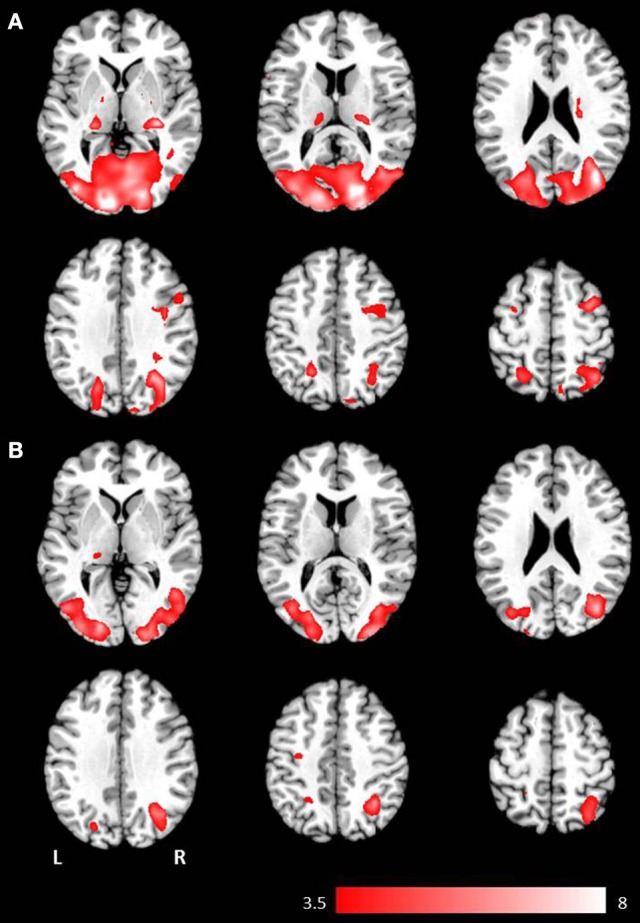
**Axial mean anatomical images overlaid with brain activation resulting from second-level GLM analysis, revealing higher brain activity for visual presentation compared to auditory presentation (*p* < 0.001, uncorrected) induced by (A) the phrases task and (B) the verb generation task**.

**Figure 2:**
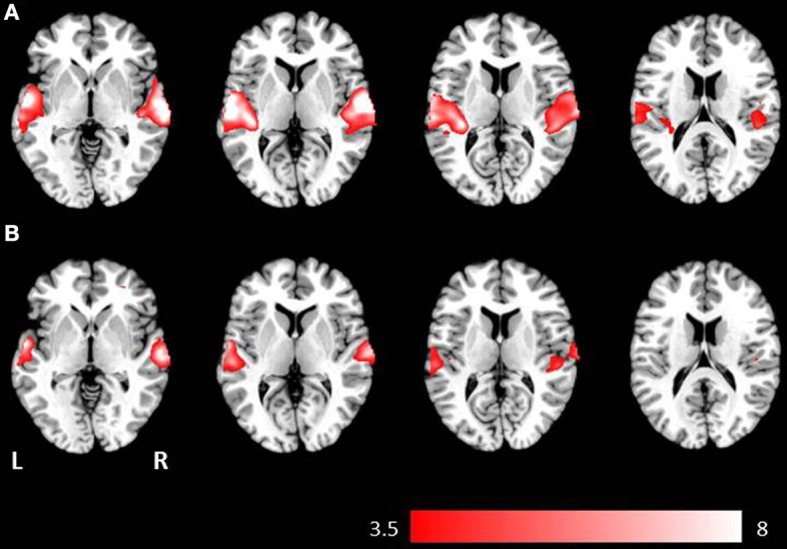
**Axial mean anatomical images overlaid with brain activation resulting from second-level GLM analysis, revealing higher brain activity for auditory presentation compared to visual presentation (*p* < 0.001, uncorrected) induced by (A) the phrases task and (B) the verb generation task**.

To map the modality-specific effects of language processing on brain activity, a two-sample *t*-test was performed at the group level. These analyses comprise the t-contrasts visual > auditory (see Figure 1) and auditory > visual (see Figure 2) computed at the group level for both investigated language paradigms. All resulting statistical parametric maps were thresholded at *p* < 0.001 (uncorrected), using a cluster extent threshold of 10 contiguous voxels.

Results for the contrast visual > auditory revealed significantly higher brain activation in the superior and inferior parietal lobule, the middle occipital gyrus, the postcentral gyrus, and in the hippocampus. For the phrases task, additional increased brain activity was obtained in the middle frontal gyrus, the precuneus, the cuneus, the precentral gyrus, and in the pallidum (see Table 1 and Figure 1B). For the verb generation task, the contrast visual > auditory evoked additional increased brain activity in the inferior temporal gyrus (see Table 1 and Figure 1A).

**Table 1:** **Significantly higher activated brain areas by visual compared to auditory presentation of the two language paradigms**.

	**Cluster size^a^**	**Anatomical label^b^**	***p*-value^c^**	**MNI coordinates**		
				***x***	***y***	***z***
**Visual > Auditory**						
Phrases	19301	Middle occipital gyrus (calcarine fissure)	<0.001	−14	−94	−4
	614	Middle frontal gyrus	<0.001	34	4	58
	337	Hippocampus	<0.001	30	−26	−2
	307	Superior parietal lobule	<0.001	−24	−56	42
	177	Hippocampus	<0.001	−26	−28	0
	63	Precuneus	<0.001	−8	−68	60
	55	Cuneus	<0.001	12	−82	44
	30	Parahippocampal gyrus	<0.001	18	−42	10
	22	Postcentral gyrus	<0.001	28	−40	32
	21	Pallidum	<0.001	−18	−4	−6
	16	Pallidum	<0.001	16	−2	−6
	16	Precentral gyrus	<0.001	−34	−2	52
	11	Inferior parietal lobule	<0.001	38	−44	36
Verb	4850	Inferior temporal gyrus	<0.001	46	−64	−12
generation	3111	Middle occipital gyrus	<0.001	−22	−92	2
	85	Cerebellum (culmen)	<0.001	38	−40	−28
	66	Inferior parietal lobule	<0.001	−30	−52	46
	29	Postcentral gyrus	<0.001	−36	−14	42
	23	Hippocampus	<0.001	−26	−28	2
	16	Superior parietal lobule	0.001	−36	−58	60

a Significantly activated clusters with 10 or more voxels.

b clusters were automatically labeled using AAL toolbox (Tzourio-Mazoyer et al., 2002).

c p < 0.001 uncorrected.

Auditory presentation (contrast auditory > visual) induced significantly increased brain activation in the superior temporal gyrus bilaterally (see Table 2 and Figure 2) for both tasks. The auditory presentation of the verb generation task revealed an additional cluster in the middle frontal gyrus (see Table 2 and Figure 2A).

**Table 2:** **Significantly higher activated brain areas by auditory compared to visual presentation of the two language paradigms**.

	**Cluster size^a^**	**Anatomical label^b^**	***p*-value^c^**	**MNI coordinates**		
				***x***	***y***	***z***
**Auditory > Visual**						
Phrases	2350	Superior temporal gyurs	<0.001	60	−12	0
	2408	Superior temporal gyrus	<0.001	−64	8	0
Verb	795	Superior temporal gyurs	<0.001	−64	−6	−2
generation	636	Superior temporal gyrus	<0.001	60	−16	−2
	25	Middle frontal gyrus	0.001	26	46	−8

a Significantly activated clusters with 10 or more voxels.

b clusters were automatically labeled using AAL toolbox (Tzourio-Mazoyer et al., 2002).

c p < 0.001 uncorrected.

**Figure 3:**
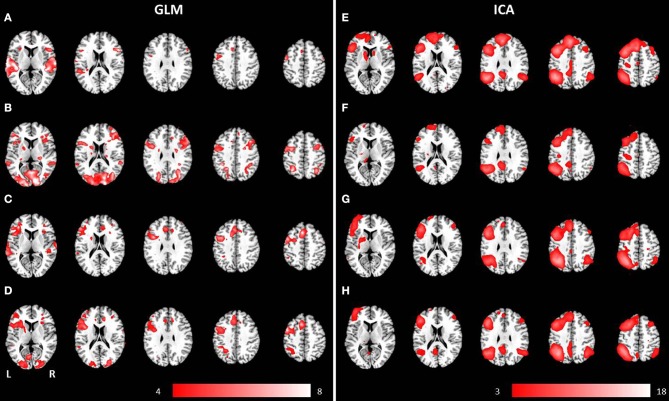
**Mean anatomical images overlaid with brain activation resulting from second-level GLM analysis and group ICA**. Results of second-level GLM analysis **(A–D)** were reported for the contrast active > baseline condition (*p* < 0.001, uncorrected) for **(A)** auditory presentation of the phrases task, **(B)** visual presentation of the phrases task, **(C)** auditory presentation of the verb generation task and **(D)** visual presentation of the verb generation task. Group ICA revealed a left lateralized language network independent from presentation modality and language paradigm. Determined networks were reported for **(E)** auditory presentation of the phrases task, **(F)** visual presentation of the phrases task, **(G)** auditory presentation of the verb generation task and **(H)** visual presentation of the verb generation task.

Auditory presentation of the phrases task evoked brain activation bilaterally in the superior temporal gyrus, the insula, the medial frontal gyrus, the inferior frontal gyrus, and the left precentral gyrus (see Figure 3A). In contrast, visual presentation of the same paradigm induced clusters of increased neuronal activation bilaterally in the superior and inferior parietal gyrus, the precentral gyrus, the lingual gyrus, the cuneus, the middle occipital gyrus, the inferior frontal gyrus, the superior temporal gyrus, the left medial frontal gyrus as well as the right middle frontal gyrus (see Figure 3B).

The auditorily presented verb generation task induced brain activation bilaterally in the medial frontal gyrus, the cingulate gyrus, the insula, the superior temporal gyrus, the left inferior frontal gyrus, the left inferior parietal lobule, and the left precentral gyrus (see Figure 3C). Visually presented stimuli also evoked neuronal activation bilaterally in the medial frontal gyrus, the cingulate gyrus and the insula. Activation of the superior temporal gyrus was obtained lateralized in the left hemisphere. Furthermore, left-sided clusters in the inferior frontal gyrus, the left inferior parietal lobule, and the precentral gyrus were larger for visual compared to auditory condition (see Figure 3D).

### Data-driven analysis (ICA)

Separate group ICA for both paradigms (phrases and verb generation) and both experiments (visual and auditory) obtained 18 components for both visually presented paradigms. For auditory presentation, group ICA revealed 24 components for the phrases task and 25 components for the auditory task. Reported activated network components only include within-brain activations.

A group language network was determined for both paradigms, independent of the presentation modality, and involved brain areas such as the inferior frontal gyrus (Broca's area), the superior temporal gyrus (Wernicke's area), the insula, the middle occipital gyrus, the precentral gyrus, and the superior frontal gyrus (see Figures 3E–H).

**Figure 4:**
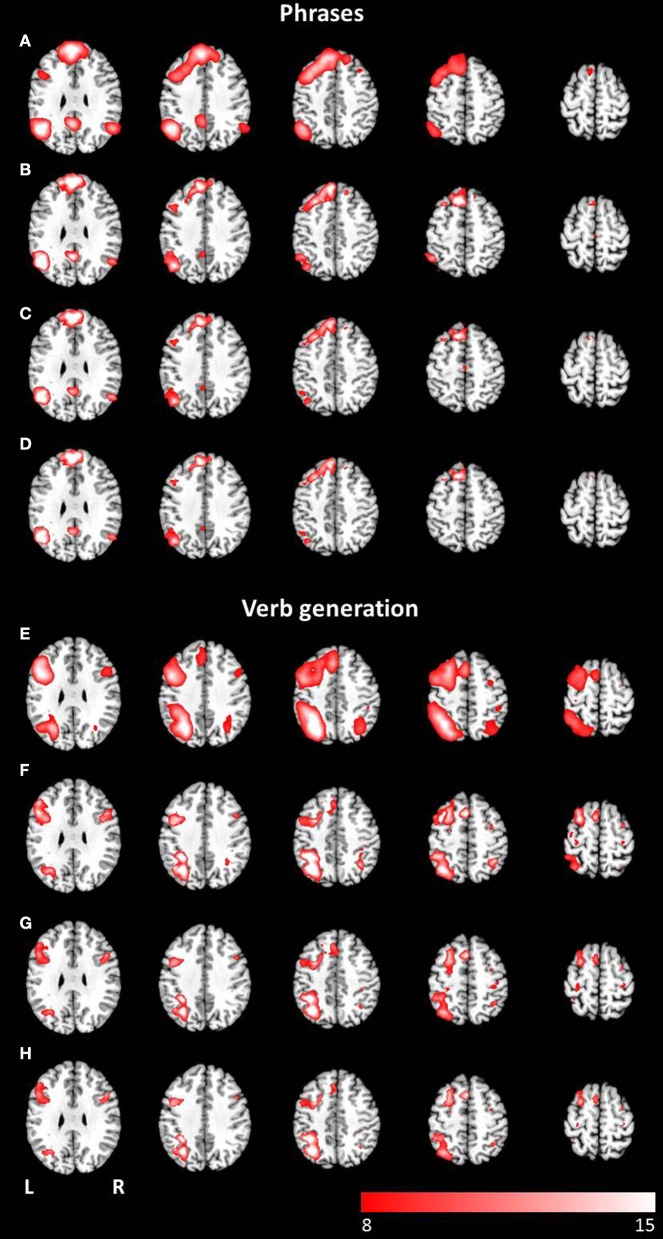
**Axial mean anatomical images overlaid with the language network, resulting from combined group ICA including auditory and visual stimulus presentation (*p* < 0.05, FWE-corrected)**. A network involving language related areas was detected for **(A)** the phrases task and **(E)** the verb generation task. The comparison of modality specific differences shows similar networks for visually **(B,F)** and auditory **(C,G)** presented language paradigms. For both paradigms the conjunction analysis of both modalities **(D,H)** revealed similar activation patterns compared to modality-specific networks.

The combined group ICA of both modalities for the phrases task and the verb generation task revealed a language and an attention network respectively. The phrases task evoked a modality independent language network, detected by performing a conjunction analysis (*p* < 0.05, FWE corrected), involving clusters in the left and right superior frontal gyrus, the left inferior frontal gyrus, the left and right angular gyrus, the left posterior cingulate cortex, the left middle temporal gyrus, and the left supplementary motor area (see Figures 4A–D). No significant differences between visual and auditory presentation were found. The verb generation task revealed a language related network including significant clusters of neuronal activation in the left and right inferior parietal lobule, the left and right inferior frontal gyrus, the left supplementary motor area, the left inferior and middle temporal gyrus, the right cerebellum, and the left and right precentral gyrus (see Figures 4E–H). Similar to the phrases task, no significant differences were detected between visual and auditory language presentation.

**Figure 5:**
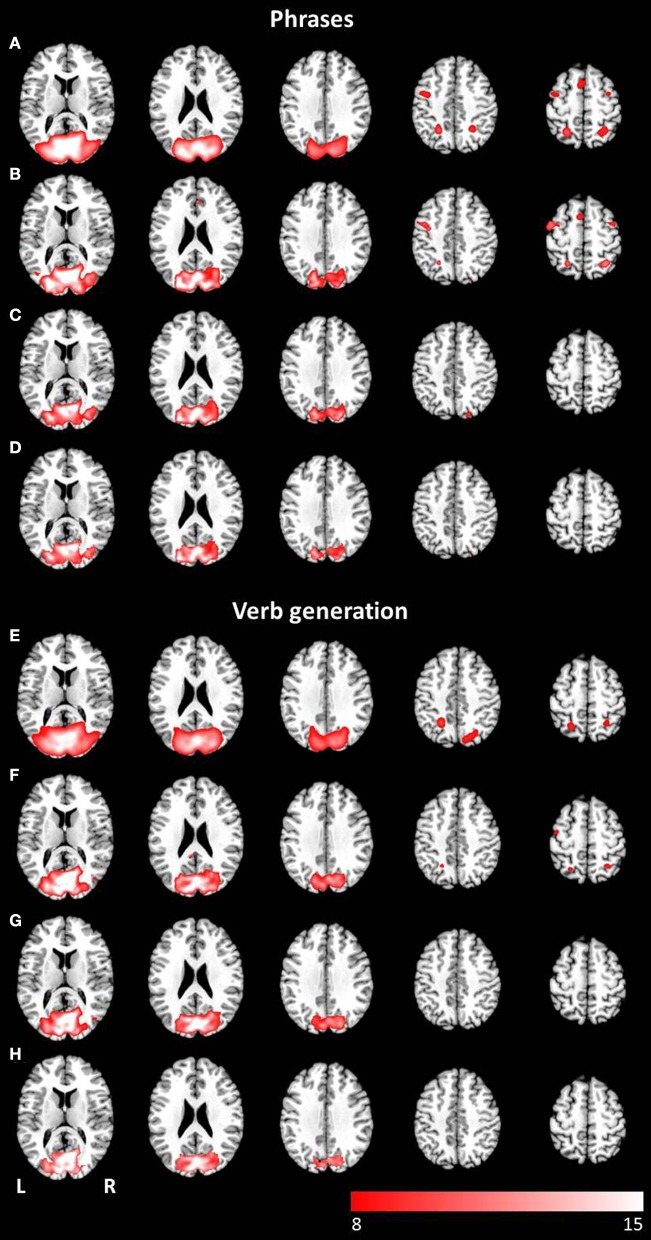
**Axial mean anatomical images overlaid with the attention-shift network, resulting from combined group ICA including auditory and visual stimulus presentation (*p* < 0.05, FWE-corrected)**. This network was determined for **(A)** the phrases task and **(E)** the verb generation task. The comparison of modality specific differences shows substantial differences for visually **(B,F)** and auditory **(C,G)** presented language paradigms. Whereas visually presented stimuli caused evoked activity in the attention-shift network, no comparable activation pattern was detected for auditory stimuli. In the conjunction analysis **(D,H)** only activation in occipital parts was found.

The combined group ICA of visual and auditory presentation of the phrases task obtained an attention network involving the left and right inferior, middle and superior occipital lobule as well as the left putamen, detected by the conjunction analysis (*p* < 0.05, FWE corrected). In addition, visual stimulus presentation revealed significant brain activation in attention related areas, involving the left superior and medial frontal gyrus, the left and right precentral gyrus, the left and right middle frontal gyrus as well as the left and right superior parietal lobule (see Figures 5A–D). No additional brain activation was obtained for auditory stimulus presentation. Based on the conjunction analysis of the two modalities for the verb generation task, an attention network involving neuronal activation bilaterally in the lingual gyrus, the calcarine gyrus, and the fusiform gyrus was detected. Visual presentation evoked additional activation in the left posterior cingulate gyrus, the left and right superior parietal lobule, and the left precentral gyrus (see Figures 5E–H). Similar to the phrases task, no additional brain activation was found for auditory presentation.

### Lateralization

**Figure 6:**
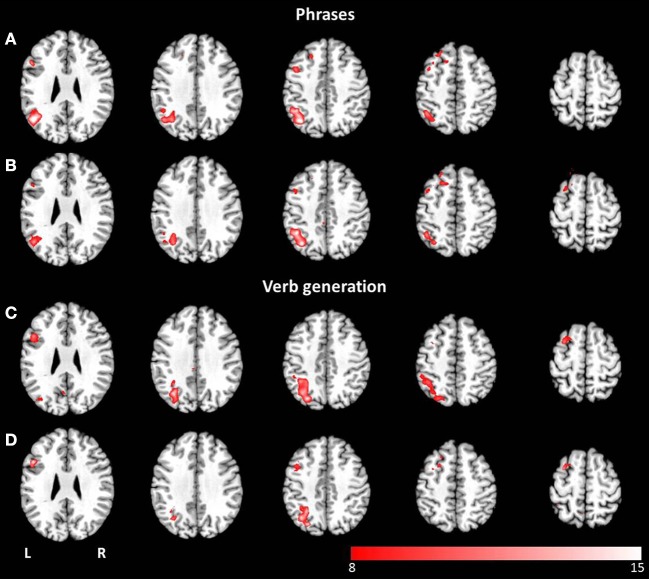
**Axial mean anatomical images overlaid with lateralized spatial maps (*p* < 0.05, FWE-corrected) for the language network resulting from combined group ICA for visual (A,C) and auditory stimulus presentation (B,D)**. No statistically significant modality-dependent differences were obtained.

Thresholded laterality maps (*p* < 0.05, FWE corrected) resulting from group ICA of the phrases task revealed significant left-sided lateralization in the inferior, middle and superior temporal gyrus, the middle and inferior frontal gyrus, the angular gyrus, and the inferior parietal lobule (see Figures 6A,B). Computation of the laterality maps resulting from the verb generation task obtained significant left lateralized brain activation in the inferior, middle and superior frontal gyrus, the inferior and superior parietal lobule, the supramarginal gyrus, the angular gyrus, the middle temporal gyrus, and the fusiform gyrus (see Figures 6C,D). For both paradigms, no significant differences were determined between visual and auditory language presentation.

## Discussion

The aim of this study was to examine modality-specific differences of language processing by comparing visually and auditorily presented language paradigms (one for language production and one for language comprehension) used in clinical practice. A combined group ICA for visual and auditory presentation revealed modality-dependent differences in identified networks. For visually presented language, an attention-shift network (Corbetta et al., 1998) was found for both paradigms. In contrast, this network was not detected for auditory presentation. These results are largely consistent with our hypothesis that visual stimulus presentation of language paradigms requires an additional attention network.

Investigating modality-dependent differences in language localization is of huge importance with respect to pre-surgical planning for which fMRI has become part of the routine procedure (Genetti et al., 2013). FMRI has been proven to be a reliable tool to determine language lateralization (Arora et al., 2009; Jones et al., 2011), and has been increasingly validated for the precise localization of language cortices (Genetti et al., 2013). The reliability of language lateralization is of particular interest in patients with left hemisphere temporal lobe epilepsy, as they have an increased likelihood of atypical right hemisphere lateralization of language processing areas (Hamberger and Cole, 2011). It is assumed that chronic epileptic activity induces a shift of language processing areas from the left to the right hemisphere (Liégeois et al., 2004; Janszky et al., 2006). Since patients prior to neurosurgery often suffer from restricted language and attention abilities, the required compliance of the patient is often lacking, which inhibits the determination of brain areas involved in language processing. Reading, in particular, may present an insurmountable challenge to patients, and therefore, paradigms for detecting language abilities are often presented auditorily for review see Dimou et al. (2013). Neuronal patterns resulting from fMRI experiments provide essential information for neuronavigation during brain surgery. Differences between auditory and visual language presentation need to be investigated in detail, as functional imaging data provide essential information for neurosurgery.

Independent of presentation modality, a language component was identified for the verb generation and for the phrases task. In clinical practice, usually both paradigms are used, as they cover different aspects of language processing. This assumption has been supported by the results of this study, showing differences in the language network between the two tasks. Investigating presentation modalities, no significant differences between auditory and visual stimulation were obtained. The involved areas of the modality-independent language network are in line with previous functional imaging results of language processing [for review see Price (2010), (2012)]. In contrast to Carpentier et al. (2001), who found higher lateralization scores for visual stimuli, the results of our study revealed no significant difference between visual and auditory stimulus presentation. Thus our findings suggest that auditory language presentation in functional imaging is an appropriate tool for lateralization, providing essential information for pre-surgical planning. However, visually presented language additionally activated an attention-shift network (Corbetta et al., 1998), which appears to be a necessary prerequisite for written language processing. A comparison of the detected attention network has shown that visual stimulus presentation evoked increased brain activation in the left superior and medial frontal gyrus, the left and right precentral gyrus, the left and right middle frontal gyrus as well as the left and right superior parietal lobule, areas related to attention (Corbetta and Shulman, 2002; Daselaar et al., 2013) and short term memory (Makuuchi and Friederici, 2013). For auditory stimulus presentation no comparable network was found. Our finding indicates that the investigator has to be aware of the individual clinical indication of functional language mapping and to select the appropriate stimulus presentation method with respect to tumor location or reorganization of networks.

Beyond modality-dependent differences, the change of spatial processing patterns induced by language and attention shifts were investigated in this study using group ICA, an already proven analysis tool for language network detection (Kim et al., 2011). The evaluation of the network components that resulted from ICA in this investigation for visual stimulus presentation revealed a network similar to the network of eye movement and attention-shift, described in Corbetta et al. (1998). For auditory stimulus presentation, this network was not detected. It is assumed that this network is responsible for covert shifts of attention, reflected by overt rapid eye movements (saccades). Moreover, these two processes appear to be not only functionally related but also share the same pathways in the brain. Although it has been shown that saccadic eye movements combined with short fixations are necessary for reading words (Reichle et al., 2003; Rayner and Reichle, 2010), the impact of saccades on word processing is still unknown (Temereanca et al., 2012).

The results of this study suggest that the performed language task as well as the presentation modality influence the detected networks. In addition, our findings indicate that not only the task itself and the way of stimulus presentation may affect the detected language processing areas. A comparison of hypothesis-driven GLM analysis and data-driven ICA showed substantial differences in resulting network patterns. Standard GLM analysis is based on the canonical hemodynamic response function (HRF) also relying on restrictive time-modeling of the stimuli. In contrast, ICA revealed highly consistent language networks independent of the language task and the modality of stimulus presentation. It is assumed that ICA is qualified to detect separate time course related networks such as attention or motor patterns (Robinson et al., 2013) and has already been shown to add additional information on processing networks (Tie et al., 2008; Schöpf et al., 2011; Frasnelli et al., 2012; Xu et al., 2013). The results of previous studies revealed that language processing areas show inter-individual variability in network patterns (Amunts et al., 2000; Rademacher et al., 2001). These individual variations in conjunction with additional stimulus-related functions such as attention or eye movements may produce imprecise language localization based on GLM analysis especially in group studies. Furthermore, a recently published study (Stoppelman et al., 2013) found significant influence of different baseline conditions on resulting language related areas using GLM analysis. Especially for the analysis of language paradigms a purely data-driven method as ICA may not only serve as an additional technique, but furthermore might be the analyzing method of choice as we were able to show that a time-locked analyzing tool, such as the GLM, was not able to reflect the spatial patterns involved in the processing of visually generated language paradigms.

Although the mapping of language processing areas using fMRI has been investigated in various studies (Carpentier et al., 2001; Arora et al., 2009; Jones et al., 2011; Genetti et al., 2013), the conductance of fMRI is sometimes problematic in clinical practice. Usually, two different language paradigms, one for language perception and another for language production, have to be performed for covering a wide range of language processing. These tasks require focused attention on the stimuli throughout the whole experiment, which is often challenging and hard to accomplish for the patient. Recently, an fMRI paradigm was presented, claiming to localize functional activation in areas for language perception and production in a single paradigm (Fedorenko et al., 2010, 2012). The validation of this paradigm in clinical practice and its effect on patient compliance should be part of further investigations.

Even though new language paradigms are developed to facilitate tasks during fMRI measurements, performing the task is still challenging the patient, due to the disabilities already mentioned previously. A promising method to overcome the substantial challenge of the patient's active participation is resting-state fMRI, a method without active task performance. Previous studies have successfully determined language networks using resting-state fMRI (Tomasi and Volkow, 2012; Kollndorfer et al., 2013; Tie et al., 2013). Although the application to pre-surgical planning has already achieved promising initial results in epilepsy surgery (Negishi et al., 2011; Morgan et al., 2012), it is still a long way from becoming part of the clinical routine (Böttger et al., 2011). In clinical practice, the development of a standardized imaging protocol for mapping language abilities, as demanded by Sunaert (2006), will be an inevitable step, as it has been shown that different resting-state conditions may influence the detected networks (Kollndorfer et al., 2013).

A potential limitation of this study is the small sample size. The influence of sample size in fMRI studies has recently been discussed controversially. Friston (2012) pointed out that statistically significant results from studies with small sample sizes are statistically valid, indicating a stronger effect than the equivalent result in a larger sample size. In contrast, some other authors (Ingre, 2013; Lindquist et al., 2013) highlight the potential pitfalls of statistical testing using small sample sizes, such as less accurate parameter estimation or less possibilities to control for confounding variables. To avoid an exceeding influence of confounding factors, we investigated a very homogeneous sample: young, healthy, right-handed subjects with comparable educational background. In addition, behavioral language data were collected to control for language ability parameters.

## Conclusion

We were able to show that the neural processing of visually presented paradigms (language perception and language production) requires an attention-shift network in addition to the commonly known language processing areas in the brain. These activation patterns were not detected for auditory stimulus presentation of the same tasks. Therefore, the way of stimulus presentation should be adjusted with respect to individual indication of functional language mapping. As the attention-shift network was restricted to visual stimuli, it is assumed that it is a basic prerequisite for reading abilities. This additional attention mechanism accompanying visually language testing may provide important information for neurosurgeons, so as to preserve language function and writing abilities to improve quality of life after surgery.

### Conflict of interest statement

The authors declare that the research was conducted in the absence of any commercial or financial relationships that could be construed as a potential conflict of interest.
